# Antibody Responses to SARS-CoV-2 among Health Care Workers in North-Eastern Tanzania

**DOI:** 10.24248/eahrj.v8i3.799

**Published:** 2025-01-30

**Authors:** Pendo Ibrahim, Felix Anthony, Happiness Mshana, Never Zekeya, Hadija Semvua, Jaffu Chilongola

**Affiliations:** a Department of Medical Biochemistry and Molecular Biology, Kilimanjaro Christian Medical University College; b Department of Clinical Trials, Kilimanjaro Clinical Research Institute; c Department of Pharmacy, Kilimanjaro Christian Medical Center; d Department of Wildlife Management, College of African Wildlife Management

## Abstract

**Background:**

Health Care Workers (HCWs) have been playing crucial role in treating patient with COVID-19. They have a higher occupational risk of contracting the disease than the general population, and a greater chance of them transmitting the disease to vulnerable patients under their care. Given the scarcity of HCWs and low COVID-19 vaccine acceptance in Africa, it is essential that HCWs are seroprotected and their exposure to COVID-19 minimized Objective: To determine IgG antibody response to SARS-CoV-2 among HCWs of a tertiary hospital in North Eastern, Tanzania.

**Methodology:**

This cross-sectional study was carried out among 273 HCWs at Kilimanjaro Christian Medical Centre (KCMC), a tertiary, zonal referral hospital in Tanzania’s North Eastern region. Stratified sampling was used to select study participants. Data were obtained from each consenting participant using a validated questionnaire. Blood samples were collected for SARS-CoV-2 IgG antibody quantification using an indirect ELISA test. RedCap software was used to manage data. Statistical analysis was done using STATA statistical software version 15 and GraphPad Prism v 9.0. A *p*-value of <0.05 was considered the cut-off for statistical significance.

**Results:**

Among 273 HCWS, 37.9 % reported receiving the COVID-19 vaccine. Except for one person, all of the participants (99.6%) had SARS-CoV-2 IgG antibody concentrations that were positive, with 64.5% of them having strong seropositivity. Cadre, sex, BMI, smoking status, adherence to recommended hand hygiene practices and COVID-19 patient interactions were significant predictors of variation of median SARS-CoV-2 antibody concentration. Age, usage of personal protective equipment, history of previously testing PCR positive for COVID-19, and total number of COVID-19 patients exposed were found to cause no statistically significant variation in median antibody concentration among participants.

**Conclusion:**

This study identified a high seroprevalence of SARS-CoV-2 antibodies among healthcare workers in the study setting, indicating significant exposure to SARS-CoV-2 virus, despite only a minority of them being vaccinated. These findings underscore the need for robust communicable disease prevention strategies including; regular screening and pathogen surveillance to better prepare for potential future pandemics. Such measures are critical to mitigating the substantial impacts on health care workers and ensuring the resilience of the healthcare system.

## BACKGROUND

Severe Acute Respiratory Syndrome Coronavirus 2 (SARS-CoV-2) is the virus responsible for causing COVID-19.^1,2^ Since its emergence in December 2019 in Wuhan China, COVID-19 has posed significant global threat, resulting in numerous deaths, economic disabilities, and social disruptions.^3–5^ COVID-19 primarily affects the respiratory system, with potential to impact other organ systems beyond the lungs.^1,6^ It was initially discovered in Wuhan, China, on December 2019.^2^ In Tanzania, the first case of COVID-19 was reported in March 2020, indicating the virus’s rapid global spread and its impact.^7^

Personnel on the front lines of treating COVID-19 patients, faced a higher occupational risk of contracting the disease than the general population. According to WHO estimates, COVID-19 resulted in approximately 115,500 deaths of healthcare workers (HCWs) worldwide.^8^ To protect this vulnerable group, WHO implemented several initiatives, including making COVID-19 vaccination a priority for HCWs.^9,10^ Nevertheless, Africa has a considerable scarcity of healthcare personnel to fulfil population demand.^11^ In Tanzania, between 0.4 and 1 HCW is available for every 1000 people.^12,13^ Despite WHO efforts, the majority of HCWs in Africa have low COVID-19 vaccine acceptance because of concerns regarding the side effects of the vaccines, safety, efficacy, short duration of the clinical trials, limited information, and social trust.^14^

Immune responses to SARS-CoV-2 are directed to the 4 main structural proteins of the virus which are; Spike (S), Envelope (E), Membrane (M), and Nucleocapsid (N) proteins.^15^ A specific humoral immune response against N and S protein has been reported and tend to persist in individuals.^16,17^ Immune responses to these proteins could result from natural immunity from infection or vaccination.^18^ However, It has also been noted that seroconversion can occur in asymptomatic people as well.^19^ The presence of neutralising antibodies against these proteins correlates with the protection against future SARS-CoV-2 infection.^20–22^

A COVID-19 seroprevalence range of 0% to 45% has been reported among HCWs in African countries by using a serological assessment of SARS-CoV-2 antibodies.^23^ A study from East Africa reported SARS-CoV-2 seroprevalence of 19.7% among HCWs in Kenya.^24^ There is a wide heterogeneity of SARS-CoV-2 seroprevalence among HCWs. This could be attributed to studies done at different timeframes during the pandemic. Additionally, the steps taken by each country’s health system to protect healthcare professionals from COVID-19 disease varied.^25^

Since the antibody response is a reliable proxy indicator of exposure to an infectious agent,^26^ monitoring SARSCoV-2 antibody response is crucial for understanding the burden of exposure to SARS-CoV-2 among the higher-risk groups such as HCWs. Assessing the concentration of SARS-COV-2 IgG antibodies among HCWs would aid in knowing not only their immunity but also exposure history to SARS-CoV-2. It will also aid in developing new strategies to protect this susceptible community from COVID-19 and future pandemic. At the time of the study’s design and conduct, no published reports on the SARS-CoV-2 IgG antibody response among HCWs in Tanzania were available. This study aimed to assess the seroprevalence of SARS-CoV-2 IgG antibodies among HCWs with different demographic profiles in North-Eastern Tanzania.

## METHODOLOGY

### Study Setting and Design

This cross sectional study was conducted from September to November, 2022 at Kilimanjaro Christian Medical Centre (KCMC), one of the four tertiary, zonal referral hospitals in Tanzania. KCMC, a 640 bed facility with 1300 healthcare workers^27^ was purposively selected due to its designation as a national centre for managing COVID-19 cases during the pandemic, and its location in the North-Eastern region of Tanzania. Kilimanjaro and Arusha regions are renowned as Tanzania’s safari capitals. The regions are popular stopovers for adventurers who are preparing to trek Mount Kilimanjaro. This makes Kilimanjaro region vulnerable to cross border transmission of infectious diseases, including SARS-CoV-2.

### Study Population

This study involved HCWs working at KCMC during the study period. Any person employed or volunteering in this setting was selected based on the definition of a HCW by WHO.^11^ If the selected HCWs did not consent to participate or donate a blood sample, they were considered as ineligible for the study and thus excluded.

### Sample Size and Sampling Technique

Since no prior data on the prevalence of SARS-COV-2 antibodies among HCWs in Tanzania were available during the study’s design, an estimate of 50% as seroprevalence of SARS COV-2 antibodies among HCWs was assumed to calculate a sufficient sample size. Using the formula by Pourhoseingholi et al^28^, and assuming an infinite population, a desired precision of 0.05, and a confidence level of 95%, a minimum sample size of 384 HCWs was computed. With a known population of 1300 HCWs at KCMC, an estimated sample size of 297 HCWs was computed using the finite population correction formula.^29^ Out of these, 273 HCWs participated in the study based on their availability. To ensure fair representation, the HCWs were stratified into 13 strata based on the different departments at KCMC. Both inpatient and outpatient HCWs were selected from each stratum. Given the demanding schedules of the HCWs, systematic recruitment within the strata was not feasible. Therefore, a convenience sample of up to 38 HCWs was selected from each stratum.

### Data Collection Procedures

HCWs who consented to participate in the study were interviewed using a study questionnaire embedded in Redcap Software installed on an Android tablet. This tool, validated by the WHO Regional Office for Africa (AFRO), was designed specifically for use in healthcare workers cohort studies on SARS-CoV-2 antibody screening.^30^ For this study, only the sections relevant to participant enrolment were utilised. This adapted questionnaire included socio demographic and clinical characteristics, information about COVID-19 vaccination history, and COVID-19 illness, occupation and community-related behaviour during the pandemic.

### Sample Collection

From each study participant, a total of 2 millilitres of blood sample through venepuncture was collected under aseptic conditions. Samples were stored in a cool box (maintained at 4–8°C using ice packs) in the field for a maximum of 3 hours before these samples were transferred to the Biotechnology Laboratory at Kilimanjaro Clinical Research Institute for serum extraction. The samples had their serum extracted instantly upon arrival at the Laboratory. For serum extraction, samples were allowed to clot then centrifuged at 1000 g for 15 minutes. After that, the serum was collected and kept frozen at negative 20°C.

### Detection of SARS-COV-2 Antibodies

IgG antibodies against SARS-CoV-2 were detected by using Generic Assays (GA) Enzyme-Linked Immuno-Sorbent assay (ELISA) for SARS-CoV-2 IgG Screening kits (MedipanGmbHGA Generic Assays GmbH, Ludwig-Erhard-Ring 3, 15827 Blankenfelde-Mahlow OT Dahlewitz, Germany). This indirect ELISA kit was a two-stage that focuses on the Spike and Nucleocapsid antigen of the SARS-CoV-2 virus detection. The reported sensitivity and specificity of these GA ELISA tests are > 98%. ^31^ Concentration results obtained from the standard curve were then interpreted as either strong positive, positive, weakly positive, borderline or negative according to the manufacturer’s cut-off value for concentrations.

### Statistical Analysis

**Figure 1:**
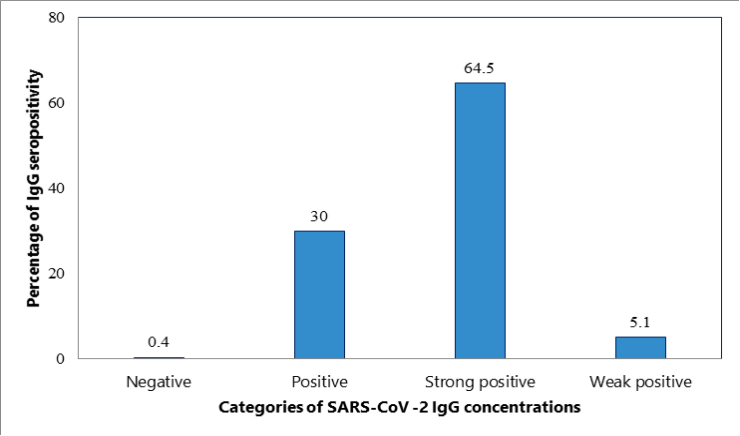
Seroprevalence of SARS-CoV-2 IgG Antibody Concentrations Among the Study participants (N=273)

**Figure 2:**
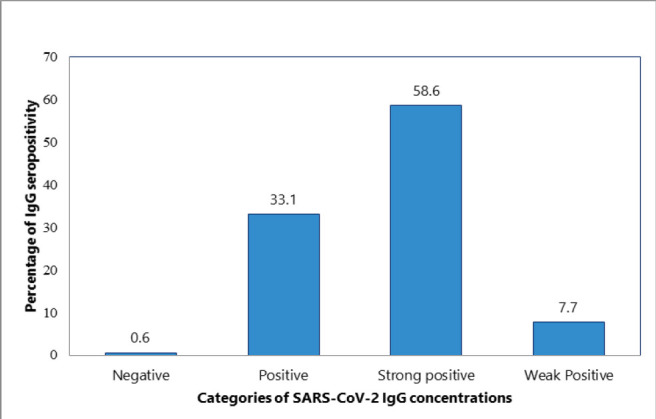
Seroprevalence of SARS-CoV-2 IgG Antibody Concentrations Among Non-Vaccinated Participants (N=169)

**Figure 3:**
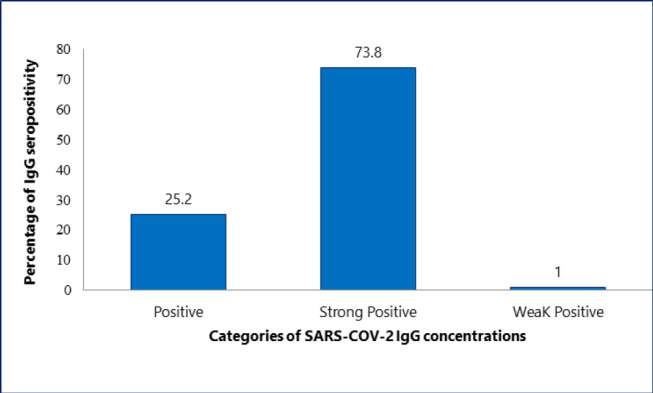
Seroprevalence of SARS-CoV-2 IgG Antibody Concentrations Among Vaccinated Participants (N=103)

STATA statistical software version 15 was used to do all statistical tests. Hence, all data from the created spreadsheet was imported to STATA. Some figures (Figure 1, 2 and 3) were generated by using GraphPad Prism v 9.5.1 Descriptive statistics was used to summarise the study participant’s baseline socio-demographic, clinical, COVID-19 exposure history and the seroprevalence of antibodies against SARS-CoV-2. After verifying that SARS-CoV-2 IgG concentration among HCWs is not normally distributed (p =0.00132 by Shapiro Wilk test), non-parametric tests were performed to compare the relationship between the exposure variables and median SARS-CoV-2 IgG concentration. The Mann–Whitney test was used to compare antibody concentrations of two independent groups. The Kruskal–Wallis test was used to compare more than two groups. A p-value of 0.05 was regarded as the cut-off for statistical significance.

### Ethical Considerations

Ethical clearance for this study was obtained from the College Research and Ethical Review Committee (CRERC) of Kilimanjaro Christian Medical University College (KCMUCo), with ethical clearance number PG61/2022. After the proposal was submitted and accepted by the ethical committee, permission from the district administration (Medical Officer, Administrative Secretary, and Executive Director), and hospital administration was sought. The questionnaire and blood samples were labelled using numbers and letters to conceal participants’ identities.

## RESULTS

### Response Rate

A total of 273 participants had their serum SARS-CoV-2 IgG concentrations determined.

### Demographic and Clinical-Exposure Characteristics of the Study Participants

Of the 273 participants tested, half were below 32 years old, with a median age of 32 (IQR: 26–44) and a male predominance of 60.4% among the total number of participants. The majority of study participants were nurses (40.5 %) and had a normal BMI (40.8%). Less than half of the study participants received the COVID-19 vaccine, and only 8.8% reported being ever tested PCR positive for COVID-19 in the past. The vast majority of participants (94.1%) stated that they had never smoked, Table 1

**Table 1:** Social demographic and Clinical Characteristics of the Study Participants (N=273)

Variable	Frequency	Percentage
Sex		
Male	165	60.4
Female	108	39.6
Age (in Years) *(n=272)		
≤ 32 years	142	52.2
> 32	130	47.8
Median (IQR)	32 (26-44)	
Cadre*(n=268)		
Medical doctor	78	29.0
Nurse	109	40.5
Allied health professionals	58	21.6
Support staff	23	8.9
BMI*(n=267)		
Underweight	6	2.3
Normal	109	40.8
Overweight	83	31.1
Obesity	69	25.8
Median (IQR)	26.4 (22.8–30.1	
Smoking status		
Stopped >1 year ago	8	2.9
Never smoked	257	94.1
Currently smoke	8	3.0
Alcohol consumption		
Stopped >1 year aeo	17	6.2
Never took alcohol	158	57.8
Currently take alcohol	98	36.0
Taking regular medication		
No	233	85.3
Yes	40	14.7
Tested PCR Positive for COVID-19*(n=272)		
No	248	91.2
Yes	24	8.8
Received COVID-19 vaccine*(n=272)		
No	169	62.1
Yes	103	37.9

* Indicates some missing values in respective variable

### Occupational and Community-Related Behaviour Factors during the Pandemic

The majority of participants (58.3%) reported interacting with COVID-19 patients. A significant proportion (56.6%) always practiced good hand hygiene as recommended, 38.9% adhered to IPC standard precautions when in contact with patients. Less than half (42.4%) consistently wore PPE based on risk assessment. Half of the participants lived in households of 3 to 5 people, and 39.5% used public transportation more than nine times a day, Table 2.

**Table 2:** Behavioural Characteristics of Study Participants (N=273)

Variable	Frequency	Percentage
Household size*(n=272)		
1–2 people	90	33.1
3–5 people	136	50.0
6–8 people	37	13.6
9+	9	3.3
Public transport		
None	75	27.5
1–2 people	68	24.9
3–5 people	19	7.0
6–8 people	3	1.1
9+	108	39.5
Stayed at least 2 meters from other people in indoor space*(n=273)		
Always	42	15.4
Did not go indoor location	31	11.4
Never	31	11.4
Often	28	10.2
Rarely	56	20.5
Sometimes	85	31.1
Hand hygiene practice* (n=265)		
Always as recommended	150	56.6
Most of the time	104	39.3
Never	3	1.1
Occasionally	8	3.0
IPC standards* (n=257)		
Always	100	38.9
I don't know what IPC standard-precaution means	22	8.6
Most of the time	97	37.7
Never	2	0.8
Occasionally	28	10.9
Rarely	8	3.1
Wearing PPE as recommended*(n=264)		
Always	112	42.4
Most of the time	102	38.6
Never	8	3.1
Occasionally	33	12.5
Rarely	9	3.4
Interactions with COVID–19 Patients* (n=264)		
No	110	41.7
Yes	154	58.3
Exposure to COVID–19 Patients*(n=247)		
1–10 Patients	169	68.4
11–50	41	16.6
51–100	18	7.3
101–500	17	6.9
> 500	2	0.8

* Indicates some missing values in respective variable

### Seroprevalence of SARS-CoV-2 IgG Antibody Concentration among the Study Participants

Nearly all participants (99.6%) tested positive for SARS-CoV-2 IgG antibody, with 64.5% exhibiting strong seropositivity (Figure 1). A comparison between vaccinated and unvaccinated individuals revealed that the majority of vaccinated participants demonstrated strong seropositivity (Figures 2 and 3).

### Socio-Demographic, Clinical, and Behavioural Characteristics Associated with Variation in Median SARS-CoV-2 IgG Concentration among Study Participants.

**Figure 4:**
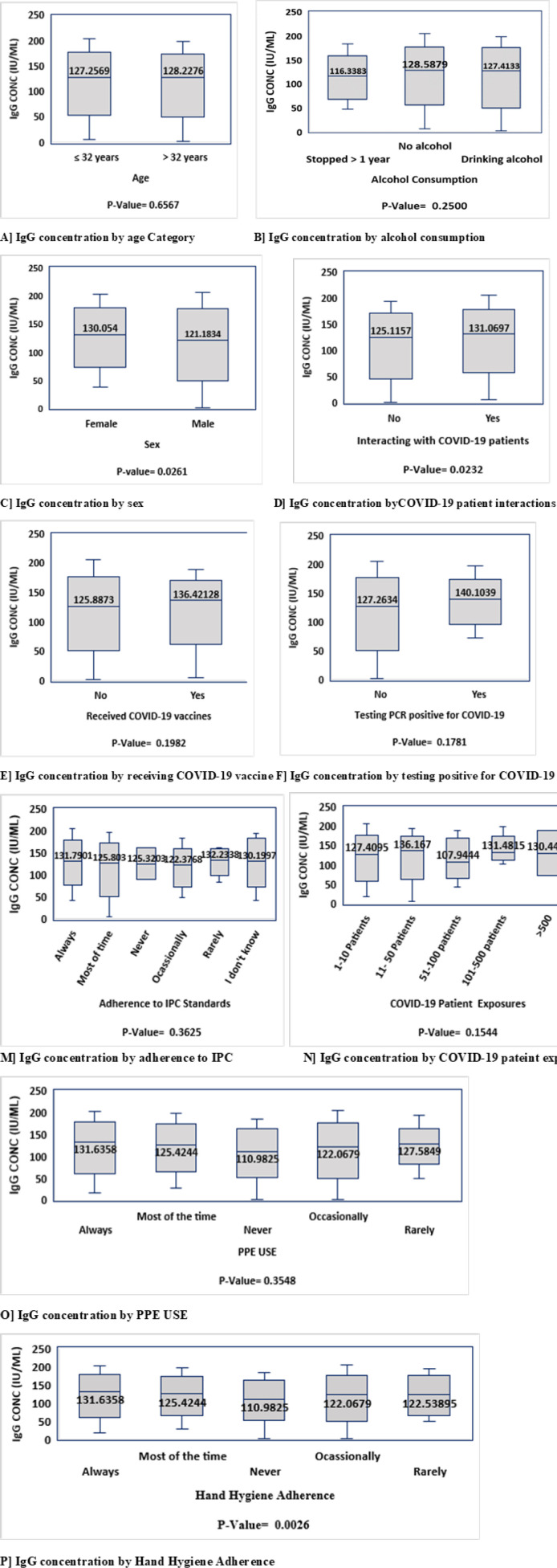
SARS COV-2 IgG Concentrations Across Different Participant Exposure Groups and Social-Behavioural Factors

Sex, BMI, smoking status, adherence to recommended hand hygiene, professional cadre, and interaction with COVID-19 patients were variables significantly influencing median SARS-CoV-2 IgG concentrations. IgG median concentration was significantly higher in females compared to males. It was found that those with obesity had significantly greater median concentrations than individuals with other BMI categories. Non-smokers showed higher SARS-CoV-2 IgG median concentrations than current smokers. Individuals who frequently adhered to recommended hand hygiene practice were found to have a significantly higher median concentration. Moreover, median concentrations were significantly greater in either individual who interacted with COVID-19 patients. Interestingly, allied health professionals had significantly higher median concentration compared to other health care workers. Other factors were assessed but did not show statistically significant differences in median SARSCOV 2 IgG concentration among participants (Figure 4).

## DISCUSSION

This study aimed to determine the IgG antibody response to SARS-CoV-2 among HCWs in KCMC. The findings revealed a remarkably high seroprevalence of 99.6% among the sampled HCWs. Notably, significantly higher median SARS-CoV-2 antibody concentrations were observed in females, allied health professionals, obese participants, HCWs who adhered to recommended hand hygiene practices, and those with frequent interactions with COVID-19 patients. This higher seroprevalence of SARS-CoV-2 antibodies among HCWs reflects the substantial level of virus exposure in their occupation and the ongoing risk of infection within the hospital. These findings align with other studies that have reported high seroprevalence of SARS-CoV-2 antibodies among HCWs.^32,33^ However, the seroprevalence observed in this study is higher than that reported in other East African countries.^34–36^ This significant discrepancy may be attributed to the level of COVID-19 precautions initially implemented in Tanzania as compared to other East African countries.

Our study findings revealed that HCWs who interacted with COVID-19 patients had significantly higher median antibody concentrations. However, it’s important to note that the number of COVID-19 patients to which a HCW was exposed to did not predict seroconversion. This confirms that while exposure to COVID-19 patients may be a significant factor in the detection of SARS-CoV-2 antibodies among HCWs, the sheer number of patients alone is not a reliable predictor of seroconversion. These results are consistent with earlier studies that demonstrated regular interaction with COVID-19 patients increases the risk of contracting SARS-CoV-2.^25,37–41^

Contrary to expectations, this study did not find any statistical differences in antibody concentrations among HCWs based on their history of testing PCR positive for COVID-19. Unlike previous studies, this study was unable to demonstrate that a prior SARS-CoV-2 infection leads to seropositivity for SARS-CoV-2 antibodies.^25,42–44^ Several factors may have influenced these results. The low percentage of healthcare workers who tested positive in this study might have contributed to the findings. Additionally, the significant decline in SARS-CoV-2 antibody levels after infection could additionally explain these results.^45^ Therefore, identifying an appropriate time interval for antibody monitoring is crucial to determine how long SARS-CoV-2 antibodies persist.

This study found that allied health professional had a higher median SARS-CoV-2 IgG concentration compared to other HCWs cadres. This indicates an increased risk of SARS-CoV-2 exposure among this group. A plausible explanation could be that allied health professionals often engage in hands-on, close-contact care with patients, including those with COVID-19. Such direct and frequent interactions likely lead to higher exposure levels. Additionally, allied health professionals may perform tasks that require prolonged presence in contaminated areas or close proximity to infected patients, further heightening their risk. These findings align with a previous observational study, that reported higher odds of seropositivity among allied health professionals compared to medical doctors.^46^ The specific reasons behind this finding will be explored in a separate study, aiming to identify strategies to better protect allied healthcare workers from the risk of acquiring communicable diseases in their work environment.

Another significant finding was the higher median IgG antibody concentration observed among HCWs who strictly adhered to recommended hand hygiene practices during the pandemic. This finding contrasts with other studies that found no association between self-reported hand hygiene adherence and SARS-CoV-2 antibody positivity among HCWs.^47^ Hand hygiene is a critical component of infection prevention practices in hospitals and reflects underlying behaviours, attitudes, and beliefs.^48^ It is a possibility that healthcare workers who adhered to recommended hand hygiene were also more likely to have received the COVID-19 vaccine. However, this hypothesis was not explored in the current study and warrants further exploration to better understand the interplay between hand hygiene practices, vaccination uptake, and SARS-CoV-2 antibody responses.

Our findings indicate that females had higher median concentrations of SARS-CoV-2 antibodies than males, supporting the theory that females tend to produce higher antibody levels after infections, potentially due to male androgens being suppressive to the immune system,^49^ exposing males to serious adverse clinical outcomes and higher mortality rates.^53,54^ Contrary to our findings, several other studies have reported higher SARS-CoV-2 antibody levels in male HCWs compared to females, often attributing this observation to behavioural differences.^35,39,50–52^

The results of this study suggests that individuals who currently smoke exhibits lower antibody response to SARSCoV-2. This may be due to the fact that smoking increases the clearance of circulating antibodies by enhancing the production of monocytes and macrophages.^55,56^ However, it is important to note that other studies have not found any association between smoking and SARS-CoV-2 antibody concentrations. These discrepancies are likely due to differences in study population characteristics, methodologies, or other confounding factors.^57,58^ Further research is necessary to clarify the relationship between smoking and antibody responses to SARS-CoV-2.

## CONCLUSION

This study reports findings of a comprehensive assessment of the IgG antibody response to SARS-CoV-2 among HCWs at KCMC in Tanzania, showing a remarkably high seroprevalence. The results pinpoint several key factors associated with higher median SARS-CoV-2 IgG antibody concentrations, including female gender, allied health professional status, obesity, adherence to hand hygiene practices, frequent interaction with COVID-19 patients, and COVID-19 vaccination. These findings underscore the substantial virus exposure among HCWs and the ongoing risk of infection within hospital settings. The notably higher seroprevalence observed in this study compared to other East African countries may reflect differences in the implementation of COVID-19 precautions adopted by other countries such as lock down contrary to those adopted in Tanzania. Additionally, while the correlation between adherences to hand hygiene and elevated antibody concentrations cannot be precisely explained by our findings, it may reflect better adherence to COVID-19 precautions, potentially including higher vaccine uptake among adhering HCWs. These findings contribute valuable insights into the factors influencing antibody responses among HCWs, offering potential directions for future research and targeted protective measures in healthcare settings.

### Recommendations

Based on the findings of this study, we recommend reinforcement and improvement of infection control measures, including stringent hand hygiene practices and the persistent use of personal protective equipment (PPE) by HCWs, to address the high seroprevalence and exposure levels. Allied health professionals, who are at increased risk, should receive additional protective measures, such as enhanced PPE and targeted training. Enhance disease outbreak preparedness by instituting regular surveillance of emerging pathogens to guide control strategies.

### Study limitations and strengths

While this study has achieved its objectives, several limitations should be noted. The scope of the study was limited in terms of coverage, as it was conducted in a single centre, a tertiary hospital, where SARS-CoV-2 virus exposure levels are likely higher than in other settings. Therefore, caution must be applied in result generalisation to broader populations. Additionally, the retrospective assessment of self-reported exposure may have introduced recall bias, potentially affecting the accuracy of exposure data. Notwithstanding these limitations, this is among the first studies reporting the seroprevalence of SARS-CoV-2 IgG antibodies among HCWs in Tanzania, providing valuable insights into exposure levels and antibody responses in this high-risk group. These findings contribute important baseline data for future research and the development of targeted interventions to protect HCWs.
